# Natriuretic Peptide Receptor-C Agonist Attenuates the Expression of Cell Cycle Proteins and Proliferation of Vascular Smooth Muscle Cells from Spontaneously Hypertensive Rats: Role of Gi Proteins and MAPkinase/PI3kinase Signaling

**DOI:** 10.1371/journal.pone.0076183

**Published:** 2013-10-14

**Authors:** Jasmine El Andalousi, Yuan Li, Madhu B. Anand-Srivastava

**Affiliations:** Department of Physiology, Université de Montréal, Quebec, Canada; University of Illinois at Chicago, United States of America

## Abstract

Vascular smooth muscle cells (VSMC) from spontaneously hypertensive rats (SHR) exhibit hyperproliferation and overexpression of cell cycle proteins. We earlier showed that small peptide fragments of cytoplasmic domain of natriuretic receptor-C (NPR-C) attenuate vasoactive peptide-induced hyperproliferation of VSMC. The present study investigated if C-ANP_4–23_, a specific agonist of NPR-C, could attanuate the hyperproliferation of VSMC from SHR by inhibiting the overexpression of cell cycle proteins and examine the underlying signaling pathways contributing to this inhibition. The proliferation of VSMC was determined by [^3^H] thymidine incorporation and the expression of proteins was determined by Western blotting. The hyperproliferation of VSMC from SHR and overexpression of cyclin D1,cyclin A, cyclin E, cyclin-dependent kinase 2 (cdk2), phosphorylated retinoblastoma protein (pRb), Giα proteins and enhanced phosphorylation of ERK1/2 and AKT exhibited by VSMC from SHR were attenuated by C-ANP_4–23_ to control levels. In addition, in vivo treatment of SHR with C-ANP_4–23_ also attenuated the enhanced proliferation of VSMC. Furthemore, PD98059, wortmannin and pertussis toxin, the inhibitors of MAP kinase, PI3kinase and Giα proteins respectively, also attenuated the hyperproliferation of VSMC from SHR and overexpression of cell cycle proteins to control levels. These results indicate that NPR-C activation by C-ANP_4–23_ attenuates the enhanced levels of cell cycle proteins through the inhibition of enhanced expression of Giα proteins and enhanced activation of MAPkinase/PI3kinase and results in the attenuation of hyperproliferation of VSMC from SHR. It may be suggested that C-ANP_4–23_ could be used as a therapeutic agent in the treatment of vascular complications associated with hypertension, atherosclerosis and restenosis.

## Introduction

Excessive vascular smooth muscle cell (VSMC) proliferation contributes to vascular remodeling that occurs in several vascular disease states including atherosclerosis, hypertension, and diabetes [1]. We and others reported earlier that VSMC from SHR exhibit exaggerated cell growth (proliferation) compared to VSMC from WKY rats [2], [3], [4]. The enhanced proliferation of VSMC from SHR was shown to be attributed to the enhanced levels of Giα proteins, because the treatment of VSMC from SHR with pertussis toxin that inactivates Giα proteins resulted in the restoration of enhanced proliferation to control WKY level [4]. In addition, the enhanced levels of endogenous vasoactive peptides including Ang II and ET-1 were also shown to contribute to the increased expression of Giα proteins and hyperproliferation of VSMC from SHR through the transactivation of EGF-R and MAP kinase signaling pathways [5], [6]. The exaggerated growth exhibited by VSMC from SHR was shown to be associated with progression from G1 to S phase in the presence of Ang II and FBS [7], [8]. In addition, the expression of cell cycle proteins from G1-phase that was upregulated in VSMC from SHR [7], [9] may also contribute to the increased growth.

Natriuretic peptides (NP) are a family of three peptide hormones termed atrial natriuretic peptide (ANP), brain natriuretic peptide (BNP), and C-type natriuretic peptide (CNP) [10], [11], [12] which are produced in mammalian hearts including humans [13]. ANP regulates a variety of physiological parameters including blood pressure, progesterone secretion, renin release, vasopressin release and endothelin release by interacting with receptors on the plasma membrane either to decrease or increase the levels of cAMP or cGMP respectively [14], [15], [16], [17], [18], [19], [20] or to affect ion channels [21].

Three subtypes of natriuretic peptide receptors (NPR): NPR-A, NPR-B and NPR-C have been reported [21]. NPR-A and NPR-B are membrane guanylyl cyclases, whereas NPR-C lacks guanylyl cyclase activity and is coupled to adenylyl cyclase inhibition through inhibitory guanine nucleotide-regulatory protein Giα [22], [23] or to activation of phospholipase C [24]. However, we showed that NPR-C-mediated decrease in cAMP levels contributes to the activation of PLC signaling and suggested a cross talk between NPR-C-mediated adenylyl cyclase and PLC signaling pathways [25].

NPR-C has a single transmembrane domain, an extracellular domain and a short 37 amino acid cytoplasmic domain or tail [26]. The cytoplasmic domain of NPR-C contains several G_i_ activator sequences which have been shown to inhibit adenylyl cyclase activity [27] and to attenuate Ang II-, endothelin-1 (ET-1)- and arginine-vasopressin (AVP)-induced increased proliferation of A10 VSMC via MAP kinase and phosphatidylinositol 3-kinase (PI3K) pathways [28]. Since VSMC from SHR exhibit enhanced proliferation, it was of interest to investigate [1] if NPR-C activation by C-ANP_4–23_ could also inhibit the enhanced proliferation of VSMC from SHR; [2] whether the antimitogenic effect of C-ANP_4–23_ is attributed to its ability to attenuate the expression of cell cycle proteins and [3] to examine the implication of MAP kinase/PI3 kinase signaling pathways which have been reported to contribute to the increased expression of Giα proteins as underlying mechanisms for the regulation of the expression of cell cycle proteins by C-ANP_4–23_.

We provided the evidence that NPR-C activation attenuates the enhanced levels of cell cycle proteins through inhibition of enhanced expression of Giα proteins and MAP kinase/PI3-kinase signaling and results in the attenuation of hyperproliferation of VSMC from SHR

## Materials and Methods

### Materials

PD98059 and Wortmannin were from Sigma (St-Louis, MO, USA). Pertussis toxin was purchased from Calbiochem (San Diego, CA, USA). Antibodies against Giα-2 (L5), Giα-3 (C-10), Cyclin D1(DCS-6), cyclin A (C-19), cyclin E (M-20), Cdk2 (D-12), Cdk4(DCS-35), phospho-specific-Ser^249?^/Thr^252?^Rb, Rb (IF8), phospho-specific –Tyr^204^-ERK1/2 antibody, polyclonal ERK1/2(C-14) antibody, polyclonal phospho-specific-Ser^473^-AKT antibody, total AKT antibody, and horseradish peroxidase-conjugated goat anti-mouse immunoglobulin were from Santa Cruz Biotechnology Inc. (Santa Cruz, DA, USA). L-(4, 5-^3^H) thymidine were from Amersham Biosciences (Baie d’Urfé, QC, Canada). C-ANP_4–23_ was purchased from Peninsula Laboratories (Belmont, CA, U.S.A.). All other chemicals were purchased from Sigma.

### Animal Treatment

Male SHR (1 week old) and age-matched Wistar-Kyoto (WKY) rats were purchased from Charles River Canada (St-Constant, Quebec, Canada) and housed at the University of Montreal for 2 days. One week-old SHR and age-matched WKY rats were injected intraperitoneally with C-ANP_4–23_ (10 nmol/kg body weight) twice per week for 6 weeks in 0.01 mol/L sodium phosphate buffer, pH 7.0, containing 0.05 mol/L NaCl. The control WKY rats and SHR received vehicle. The blood pressure was monitored twice a week by tail-cuff method without anesthesia. At the end of the 8^th^ week, after taking the blood pressure, the rats were euthanized by decapitation. The aorta were dissected out and used for cell culture. All the animal procedures used in the present study were approved by the Comité de Déontologie de l'Expérimentation sur les Animaux (CDEA) of the University of Montreal (#99050). The investigation conforms with the 'Guide for the Care and Use of Laboratory Animals' published by the US National Institutes of Health (NIH Publication No.85-23, revised 1996).

### Cell culture and incubation

VSMC from SHR and their age-matched WKY rats were cultured from aortas as described previously [29]. The purity of the cells was checked by immunofluorescence technique using α-actin as described previously [30]. These cells were found to contain high levels of smooth muscle-specific actin. The cells were plated in 75 cm^2^ flasks and incubated at 37^o^C in 95% air and 5% CO_2_ humidified atmosphere in Dulbecco’s modified Eagle’s medium (DMEM) (with glucose, L-glutamine and sodium bicarbonate) containing antibiotics and 10% heat-inactivated fetal bovine serum (FBS). The cells were passaged upon reaching confluence with 0.5% trypsin containing 0.2% EDTA and utilized between passages 3 and 12. Confluent cells were then starved by incubation for 3 h in DMEM without FBS at 37°C to reduce the interference by growth factors present in the serum. The cells were then incubated in the absence or presence of various concentrations of C-ANP_4–23_, pertussis toxin (100 ng/ml), PD98059 (10 µM) or wortmannin (10 µM), for 16 h. After incubation, the cells were washed three times with PBS and lysed in 30 µl of buffer (25 mM Tris-HCl, pH 7.5, 25 mM NaCl, 1 mM Na orthovanadate, 10 mM Na fluoride, 10 mM Na pyrophosphate, 2 mM ethylene, bis(oxyethylenenitrolo)tetracetic acid 2 mM ethylenediamine tetracetic acid, 1 mM phenylmethylsulfonyl fluoride, 10 µg/ml aprotinin, 1% Triton X-100, 0.1% sodium dodecyl sulphate (SDS), and 0.5 µg/ml leupeptin) on ice. The cell lysates were centrifuged at 12,000 g for 5 min at 4°C. Protein concentration was measured with the Bradford assay[31].

### Western blot analysis

The levels of Giα-2, Giα-3, cell cycle proteins, ERK1/2, pAKT(Ser473) were determined by Westernblotting using specific antibodies as described previously [32]. Equal amounts of protein (30µg) were subjected to 10% SDS-polyacrylamide gel electrophoresis (SDS-PAGE), transferred to nitrocellulose membranes and incubated with the respective primary antibodies: cyclin D1 (DCS-6), cyclin A (C-19),cyclin E (M-20), cdk4 (DCS-35), cdk2 (D-12), p-Rb (Ser 249/Thr 252), Rb (IF8), p-Akt1/2/3(Ser 473)-R, Akt1/2/3 (H-136), ERK 2 (C-14) and p-ERK1/2(Try204). The antibody-antigen complexes were detected by second antibody, and protein bands were visualized by enhanced-chemiluminescence Western-blotting detection reagents from Santa Cruz Biotechnology. Quantitative analysis of specific bands was performed by densitometric scanning of the autoradiographs with an enhanced laser densitometer (LKB Ultroscan XL, Pharmacia, Dorval, Quebec, Canada) and quantified by using gel-scan XL evaluation software (version 2.1) from Pharmacia.

### [Methyl-3H]thymidine incorporation

DNA synthesis was evaluated by incorporation of [^3^H] thymidine into cells. Subconfluent VSMC from SHR and WKY rats were plated in 6-well plates for 24 h and were serum deprived for 3 h to induce cell quiescence. The cells were then incubated with NSC 625987, NU2058, C-ANP_4–23_. [^3^H] thymidine (1 µCi) was added and further incubated for 4 h before the cells were harvested. The cells were rinsed twice with ice-cold PBS and incubated with 5% trichloroacetic acid for 1 h at 4°C. After being washed twice with ice-cold water, the cells were incubated with 0.4 N sodium hydroxide solution for 30 min at room temperature, and radioactivity was determined by liquid scintillation counter.

### Statistical analysis

Results are expressed as mean ± SEM Comparisons between groups were made with ANOVA in conjunction with the Newman–Keuls test. Results were considered significant at a value of *P*<0.05.

## Results

### C-ANP_4–23_ decreases the enhanced proliferation of VSMC from SHR

**Figure 1:**
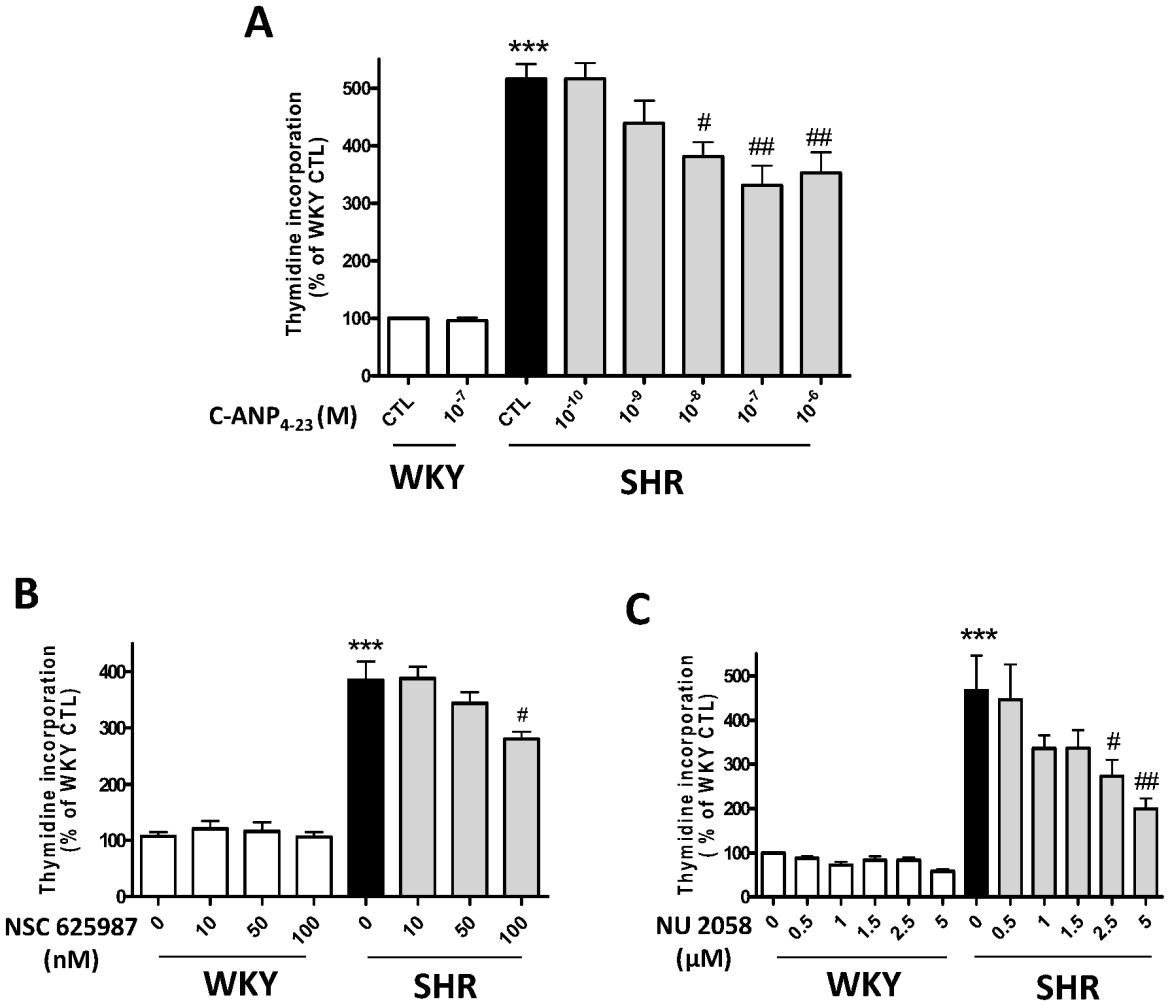
Effect of C-ANP_4–23,_ NSC 625987and NU2058 on proliferation of Vascular smooth muscle cells (VSMC) from SHR and WKY rats. VSMC from SHR and WKY rats were incubated in the absence (control) or presence of various concentrations of C-ANP_4–23_ (A), NSC 625987 (B) or NU2058 (C) for 16 h. Thymidine incorporation was determined as described in “Methods”. Results are expressed as % of WKY CTL, taken as 100%. Values are means ± SE of 5 separate experiments. *** *P*<0.01, ***P<0.001 vs WKY CTL, #*P*<0.05, ##*P*<0.01 vs SHR CTL.

We have earlier shown that small cytoplasmic domain peptides of NPR-C attenuated the enhanced proliferation of VSMC induced by vasoactive peptides in A10 VSMC [28]. In order to investigate if NPR-C activation could also attenuate the enhanced proliferation of VSMC from SHR, the effect of various concentrations of C-ANP_4–23_ on DNA synthesis was examined in VSMC from SHR and WKY rats. Results shown in Figure 1A indicate that C-ANP_4–23_ attenuated the enhanced proliferation (by about 400%) of VSMC from SHR in a concentration-dependent manner. At the optimal concentration of 10^−7^ M, the augmented proliferation of VSMC from SHR was decreased by about 50%.

### Role of Cyclin D/cyclin D kinase in enhanced proliferation of VSMC from SHR

To investigate the implication of cell cycle components in the hyperproliferation of VSMC from SHR, we examined the effect of selective inhibitors of cdk4/cyclin D1 complex (NSC 625987) and cdk2 (NU2058) on DNA synthesis in VSMC from SHR and WKY rats. Results shown in Figure 1B & C indicate that both the inhibitors NSC 625987 (B) and NU2058 (C) attenuated the enhanced proliferation of VSMC from SHR by about 35 and 60% respectively, whereas these inhibitors did not have any significant effect on the proliferation of VSMC from WKY rats.

### Effect of in vivo treatment of C-ANP_4–23_ on DNA synthesis in VSMC from SHR

**Figure 2:**
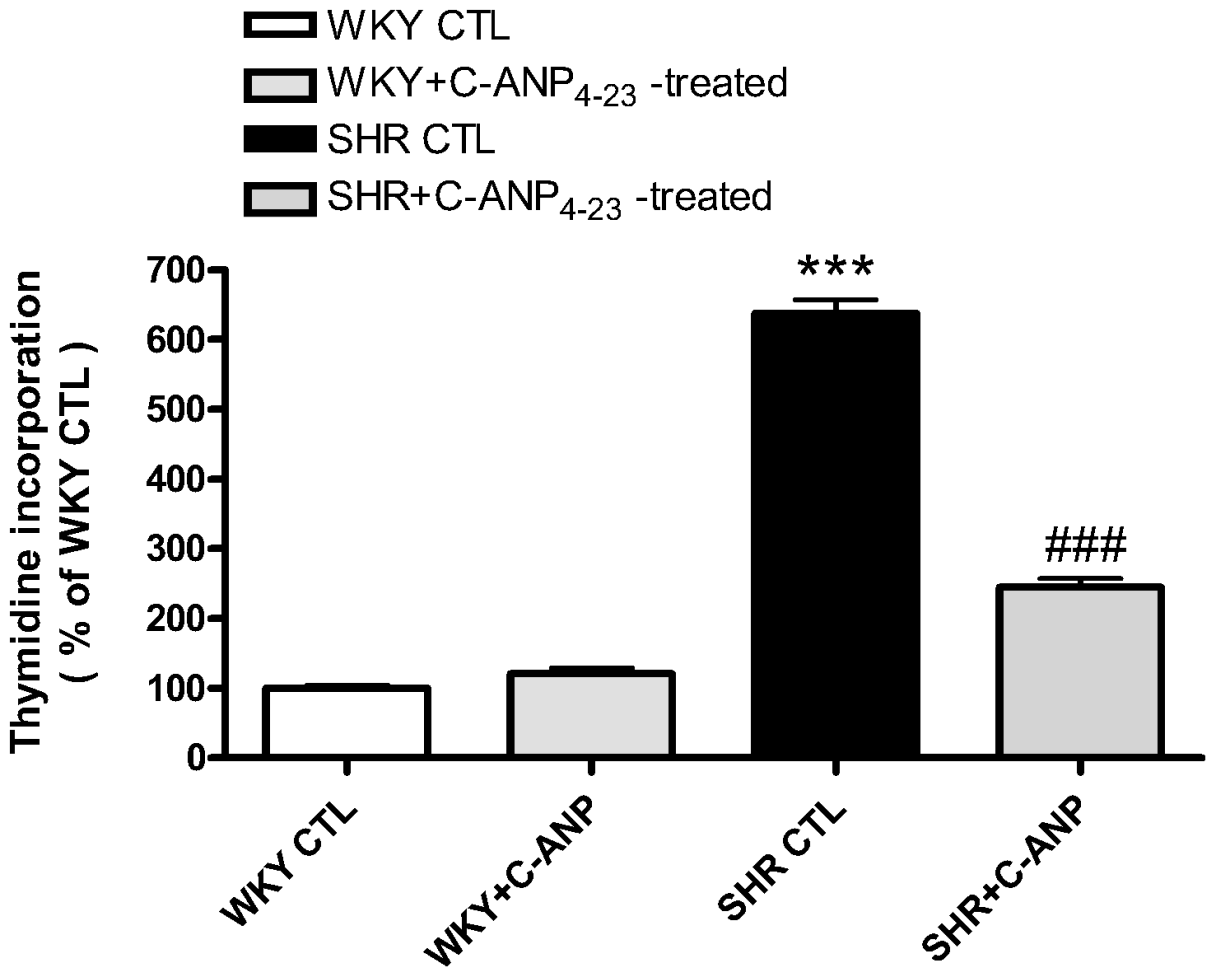
Effect of in vivo treatment of C-ANP_4–23_ on DNA synthesis in VSMC from 8 week-old SHR and age-matched WKY rats. One week old SHR and age matched WKY rats (control) were injected intraperitoneally with C-ANP_4–23_ (10 nmol/Kg of body weight) twice weekly up to 8 weeks as described in “Methods”. After eight weeks of treatment, the rats were sacrificed and aortic VSMC from SHR and age-matched WKY (control groups) and C-ANP-_4–23_-treated groups were cultured and thymidine incorporation was determined as described in “Methods”. Results are expressed as % of WKY CTL, taken as 100%. Values are means ± SE of 3 separate experiments.. *** P<0.001 vs WKY, ^###^ P<0.001 vs SHR.

We earlier showed that in vivo treatment of SHR with C-ANP_4–23_ attenuated the development of high blood pressure [33]. We further investigated the effect of in vivo treatment of C-ANP_4–23_ on DNA synthesis in VSMC from SHR and WKY rats and the results are shown in Figure 2. C-ANP_4–23_ treatment of SHR attenuated the enhanced proliferation of VSMC by about 60%, whereas it did not have any effect on DNA synthesis in VSMC from WKY rats.

### C-ANP_4–23_ attenuates the expression of cell cycle proteins in VSMC from SHR

**Figure 3:**
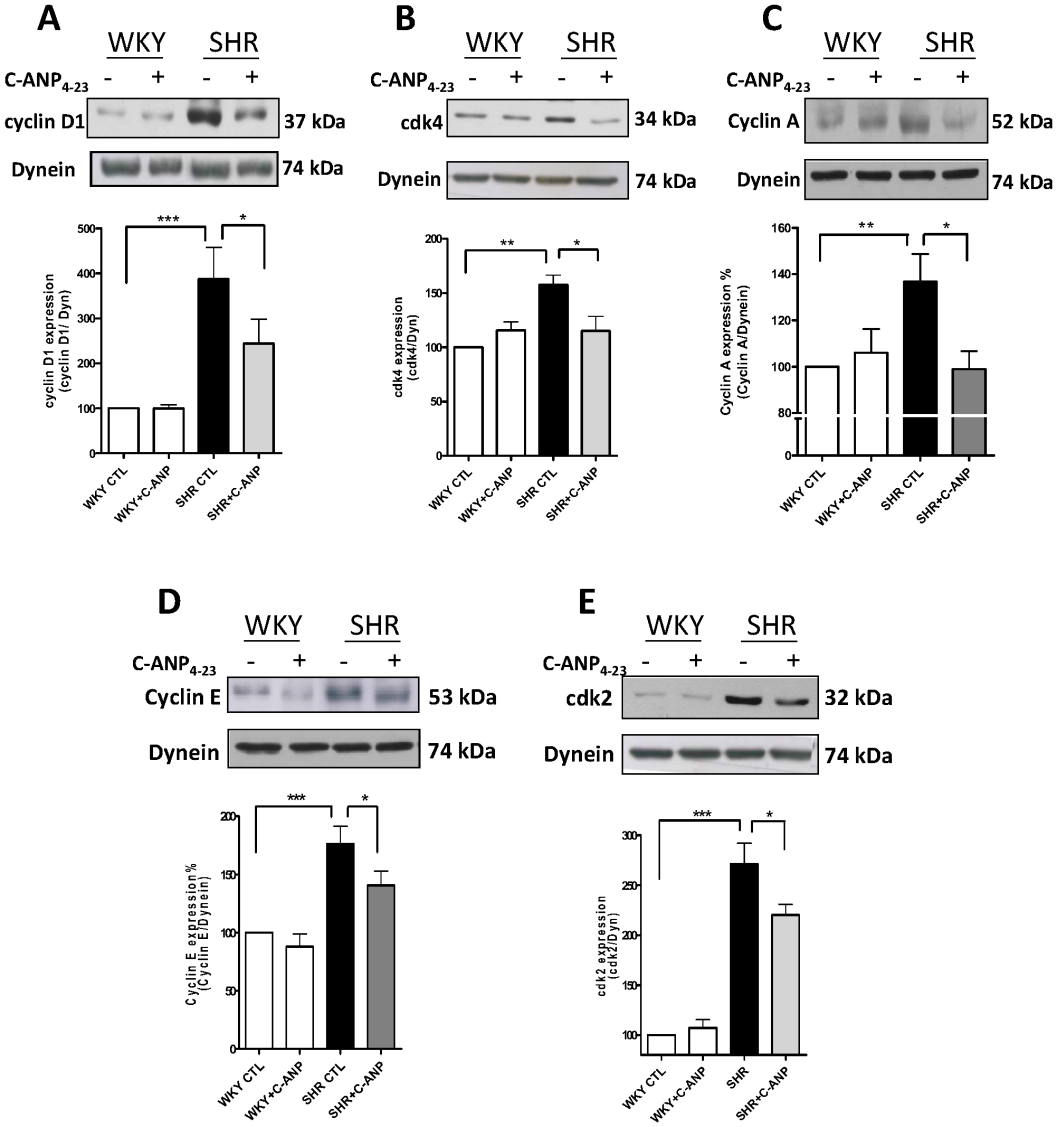
Effect of of C-ANP_4–23_ on the expression of cell cycle components in vascular smooth muscle cells (VSMC) from SHR and WKY rats. VSMC from SHR and WKY rats were incubated in the absence (control) or presence of C-ANP_4–23_ (10^−7^M) for 16 h. The cell lysates were prepared and used for Western blotting using specific antibodies against cyclin D1 (A), cdk4 (B), cyclin A (C), cyclin E (D) and cdk2 (E) as described in “Methods”. Results are expressed as % of WKY CTL taken as 100%. Values are means ± SE of 5 separate experiments. *P<0.05, **P<0.01, ***P<0.001.

**Figure 4:**
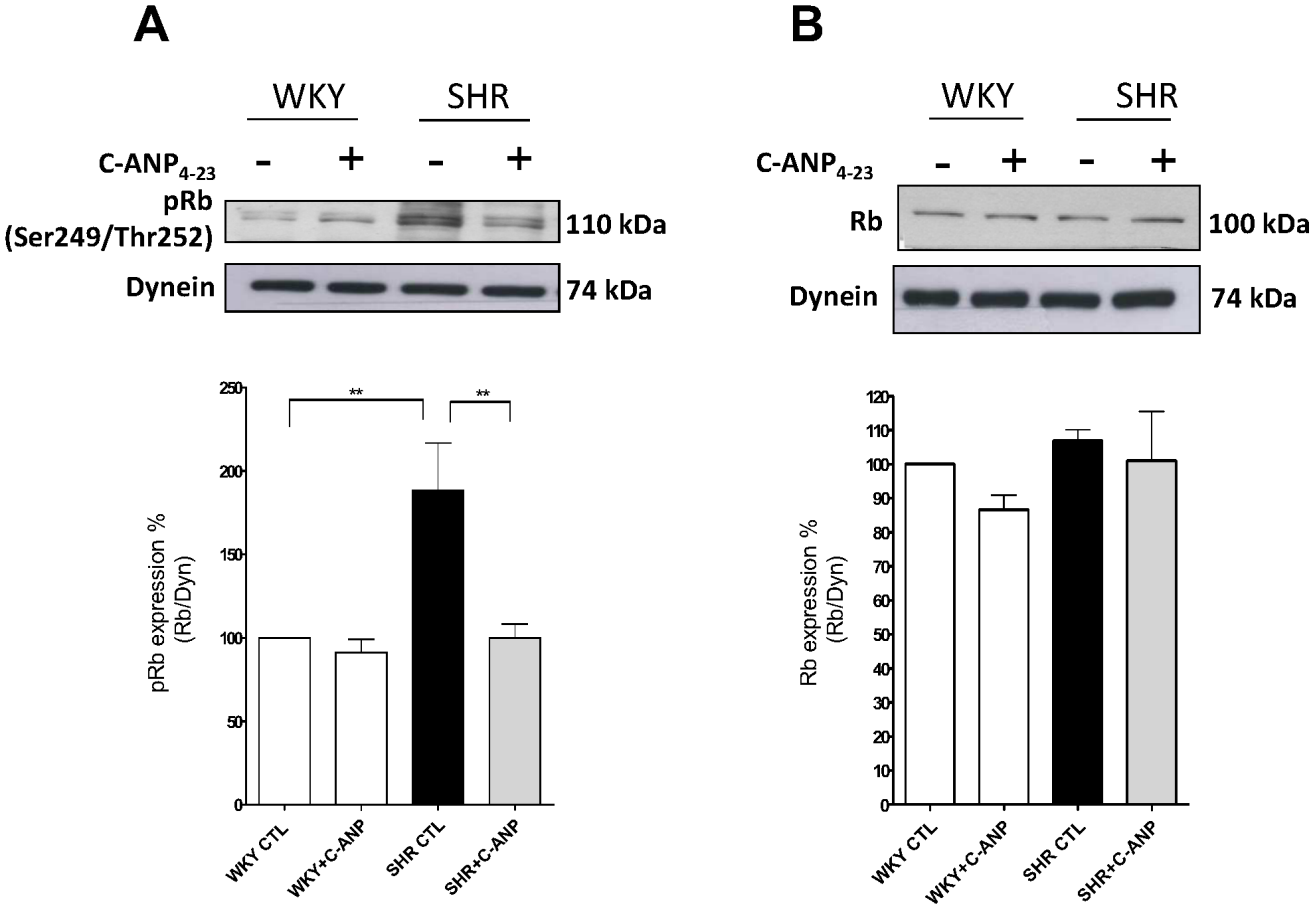
Effect of C-ANP_4–23_ on the expression of phosphorylated retinoblastoma protein (pRb) and retinoblastoma protein (Rb) in vascular smooth muscle cells (VSMC) from SHR and WKY rats. VSMC from SHR and WKY rats were incubated in the absence (control) or presence 10^-7^M of C-ANP_4–23_ for 16 h. The cell lysates were prepared and used for Western blotting using specific antibodies against pRb (A) and Rb (B) as described in “Methods”. Results are expressed as % of WKY CTL taken as 100%. Values are means ± SE of 5 separate experiments. **P<0.01.

To investigate the mechanism by which NPR-C activation inhibits the enhanced proliferation of VSMC from SHR, we examined the effect of C-ANP_4–23_ on the expression of the cell cycle proteins. As shown in Figure 3, the expression of cyclin D1(A) and cdk4 (B) was significantly enhanced by about 275% and 60% respectively in VSMC from SHR as compared to WKY and C-ANP_4–23_ attenuated the enhanced expression of cyclin D1 by about 45% whereas it completely abolished the enhanced expression of cdk4. In addition, the expression of cyclin A (C), cyclin E (D) and cdk2 (E) protein was also significantly enhanced by about 40%, 75% and 170% respectively in VSMC from SHR compared to WKY and C-ANP_4–23_ treatment completely abolished the enhanced expression of cyclin A to control levels whereas the enhanced expression of cyclin E and cdk2 was attenuated by about 45% and 30% by C-ANP_4–23_. These results suggest that NPR-C activation decreased the expression of cell cycle components from G1-S phase. Furthermore, the expression of phosphorylated retinoblastoma protein (pRb) (Figure 4A) but not of Rb (Figure 4B) was enhanced by about 35% in VSMC from SHR which was also completely abolished by C-ANP_4–23_. Since the levels of phosphorylation of pRb protein is a marker of cell cycle progression, this suggests that NPR-C activation leads to an inhibition of the cell cycle progression.

### Role of Giα proteins in the enhanced expression of cell cycle proteins in VSMC from SHR

**Figure 5:**
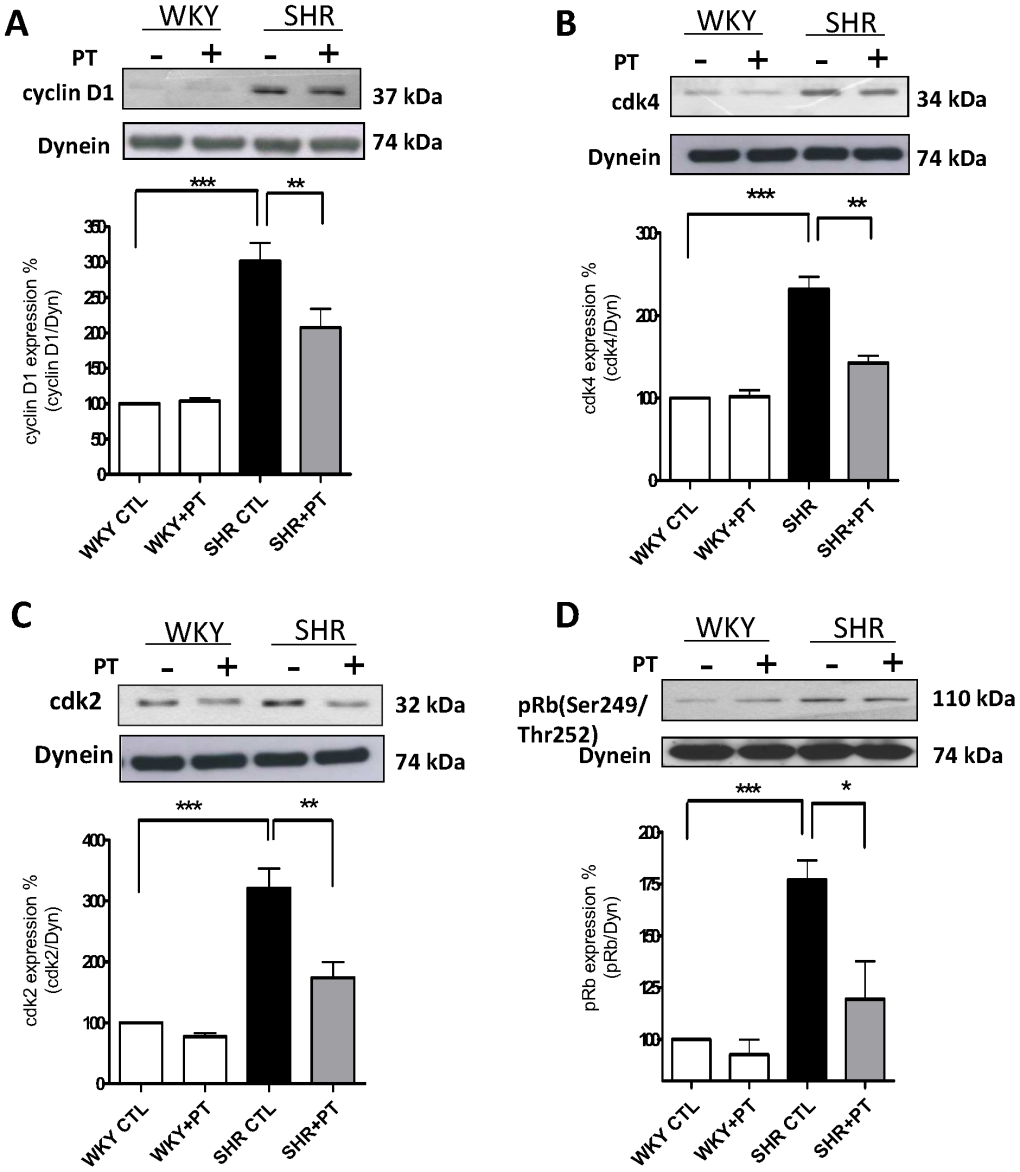
Effect of pertussis toxin on the expression of cell cycle components from G1-phase in vascular smooth muscle cells (VSMC) from SHR and WKY rats. VSMC from SHR and WKY rats were incubated for 16 h in the absence (control) or presence of pertussis toxin (PT, 10 ng/ml). The cell lysates were used for Western blotting as described in “Methods”. Results are expressed as % of WKY CTL, taken as 100%. Values are means ± SE of 5 separate experiments. *P<0.05, **P<0.01 ***P<0.001.

We have previously shown the implication of enhanced levels of Giα proteins in the enhanced proliferation of VSMC from SHR [4]. To investigate if the enhanced expression of Giα proteins contribute to the overexpression of cell cycle proteins in VSMC from SHR, the effect of pertussis toxin (PT) treatment on the levels of cell cycle proteins was examined in VSMC from SHR and WKY rats. Results shown in Figure 5, demonstrate that the treatment of VSMC from SHR with pertussis toxin, attenuated the enhanced expression of cyclin D1 (A), cdk2 (C) and pRb (D) or Rb (D) proteins by about 50-65% in VSMC from SHR. In addition, the expression of these proteins was not altered by PT in VSMC from WKY rats.

### C-ANP_4–23_ decreases the enhanced expression of Giα proteins in VSMC from SHR

**Figure 6:**
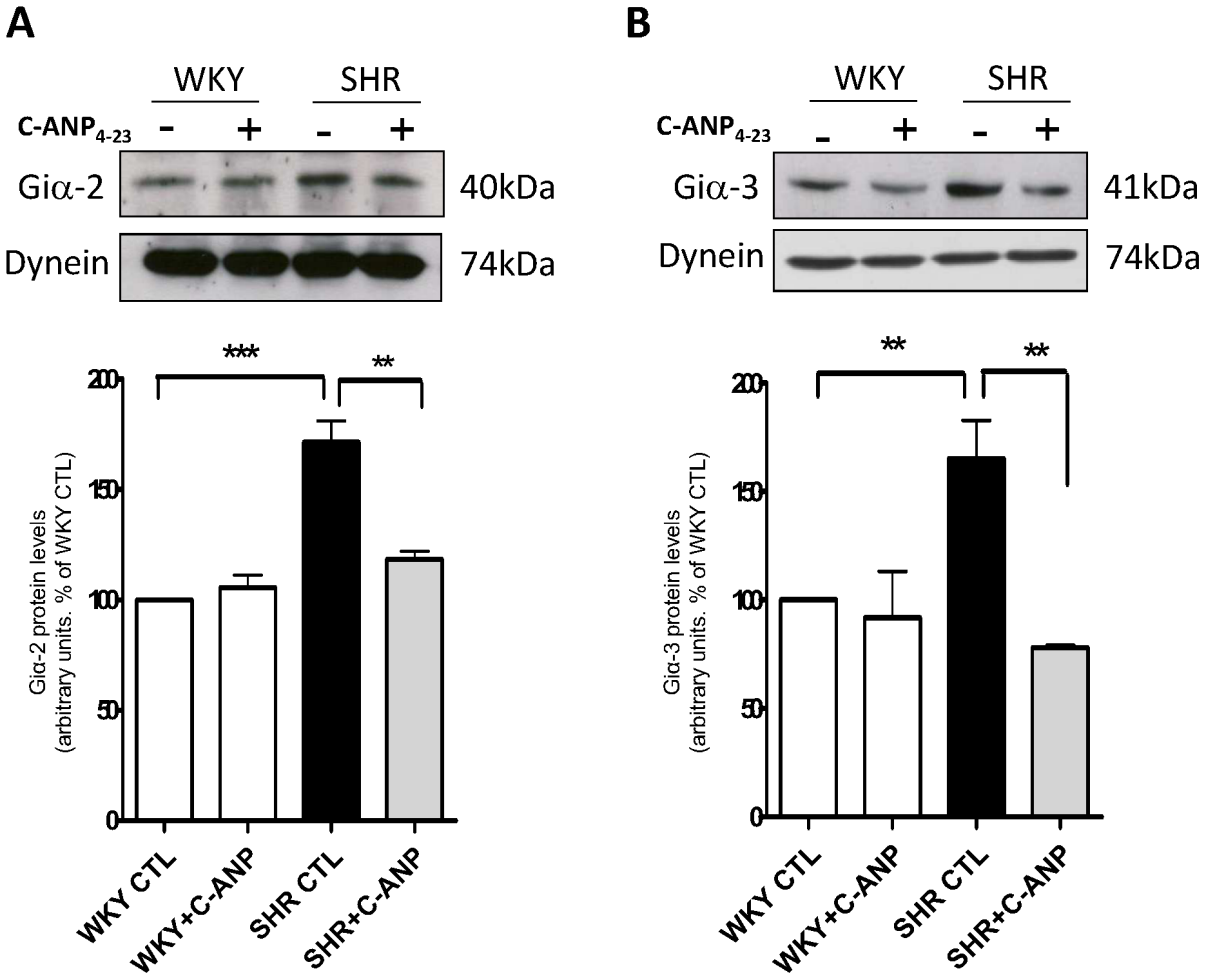
Effect of C-ANP_4–23_ on the expression of Giα-2 and Giα-3 proteins in vascular smooth muscle cells (VSMC) from SHR and WKY rats. VSMC from SHR and WKY rats were incubated in the absence (control) or presence 10^-7^M of C-ANP_4–23_ for 16 h. The cell lysates were prepared and used for Western blotting using specific antibodies against Giα-2 (A) and Giα-3 (B) as described in “Methods”. Results are expressed as % of WKY CTL, taken as 100%. Values are means ± SE of 5 separate experiments. **P<0.01, ***P<0.001.

To further examine if C-ANP_4–23_-induced decreased expression of cell cycle components in VSMC from SHR is also attributed to its ability to decrease the expression of Giα proteins, the effect of C-ANP_4–23_ on the levels of Giα proteins was determined and the results are shown in Figure 6. C-ANP_4–23_ like PT also attenuated the enhanced expression of Giα-2 (A) as well as Giα-3 (B) proteins in VSMC from SHR towards WKY control levels suggesting that C-ANP_4–23_-induced decreased expression of Gi proteins may contribute to the attenuation of the enhanced expression of cell cycle components in VSMC from SHR.

### Role of MAP kinase and PI3kinase in enhanced expression of cell cycle proteins in VSMC from SHR

**Figure 7:**
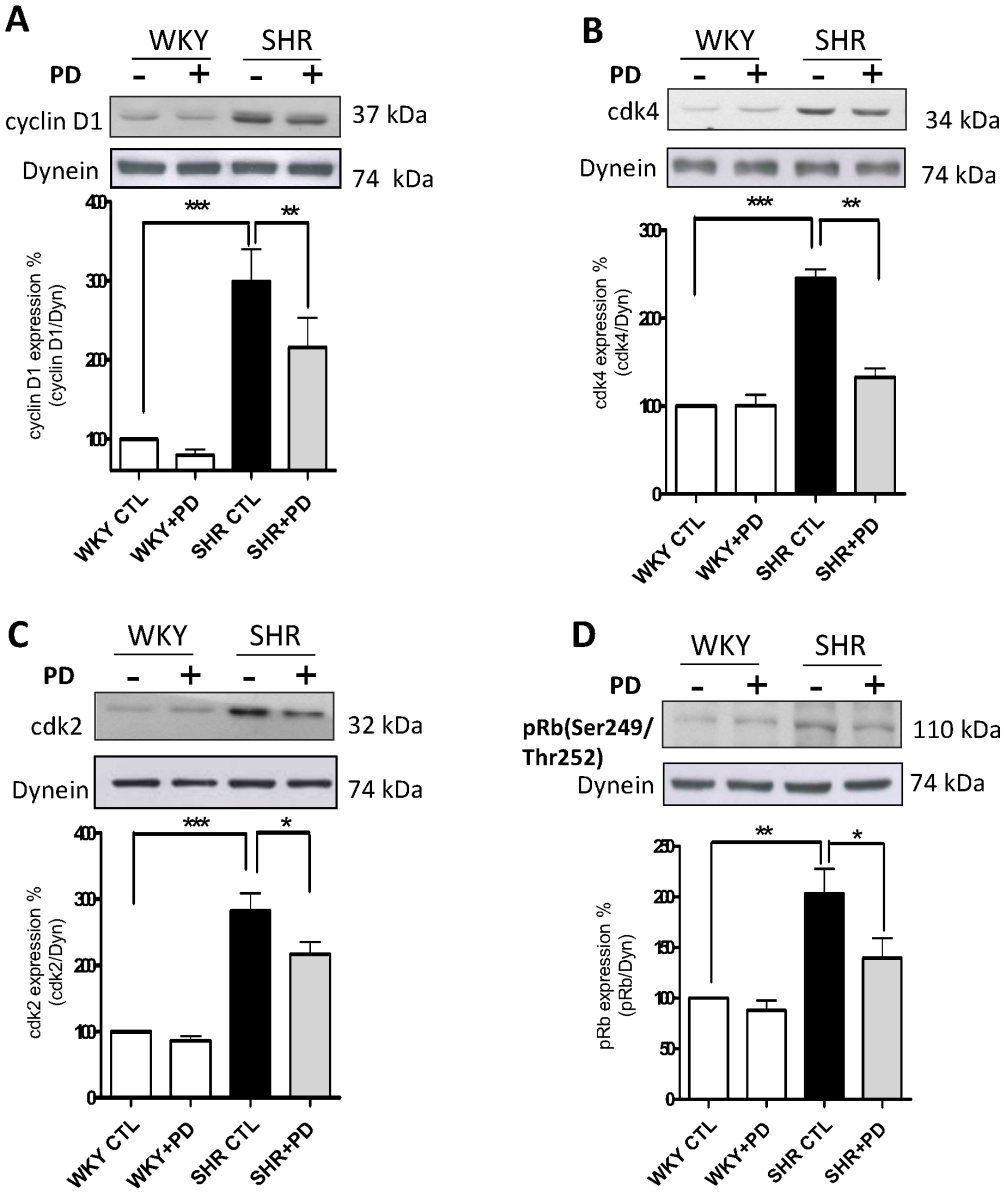
Effect of PD 98059 on the expression of cell cycle components from G1-phase in vascular smooth muscle cells (VSMC) from SHR and WKY rats. VSMC from SHR and WKY rats were incubated for 16 h in the absence (control) or presence of 10 µM PD 98059 (PD). The cell lysates were prepared and used for Western blotting using specific antibodies against cyclin D1 (A), cdk4 (B), cdk2 (C) and pRB (D) as described in “Methods”. Results are expressed as % of WKY CTL taken as 100%. Values are means ± SE of 5 separate experiments. *P<0.05, **P<0.01, ***P<0.001.

**Figure 8:**
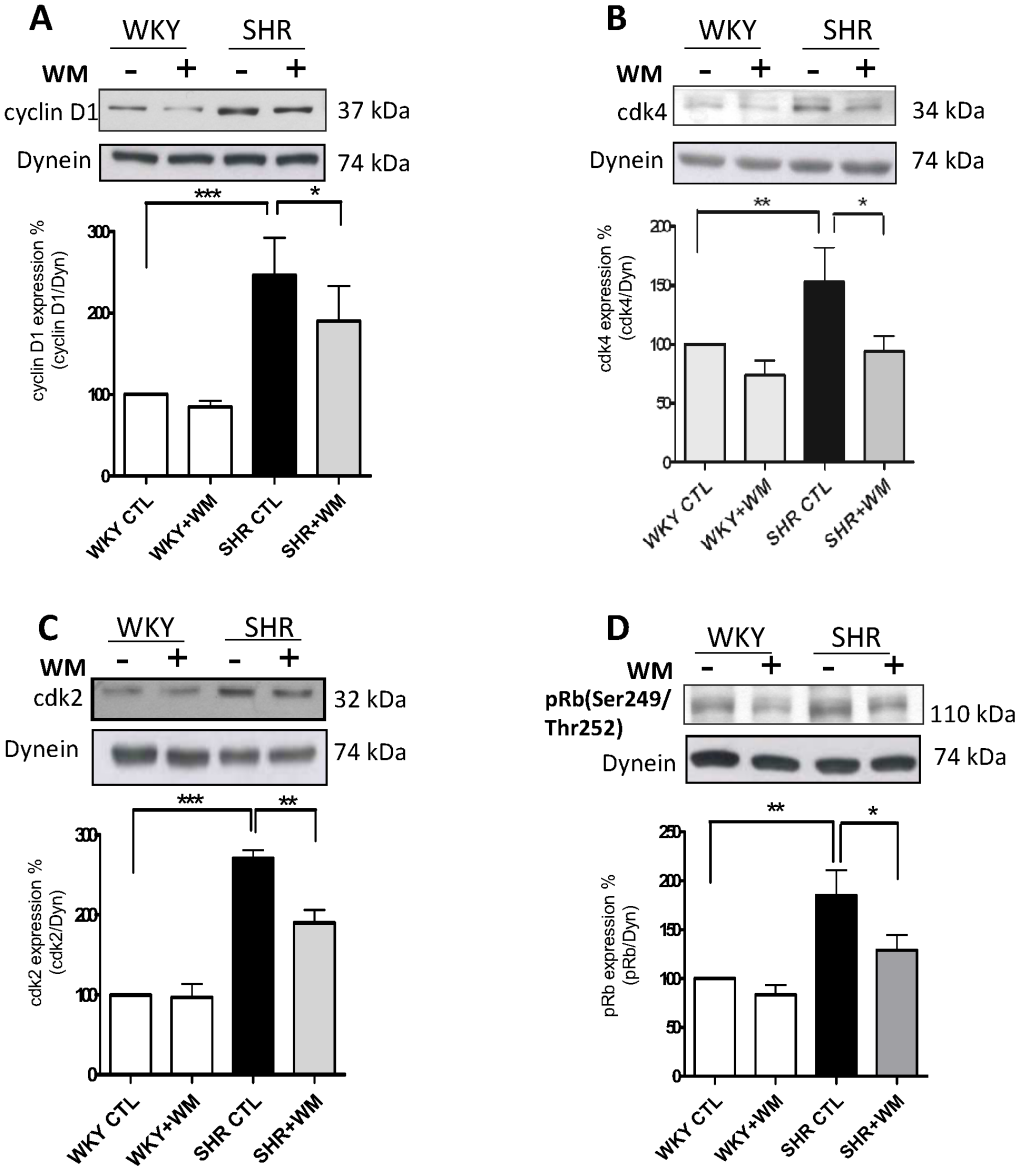
Effect of wortmannin on expression of cell cycle components from G1-phase in vascular smooth muscle cells (VSMC) from SHR and WKY rats. VSMC from SHR and WKY rats were incubated for 16 h in the absence (control) or presence of 1 µM wortmannin WM). The cell lysates were prepared and used for Western blotting using specific antibodies against cyclin D1 (A), cdk4 (B), cdk2 (C) and pRB (D) as described in “Methods”. Results are expressed as % of WKY CTL taken as 100%. Values are means ± SE of 5 separate experiments. *P<0.05, **P<0.01, * **P<0.001.

We previously showed that VSMC from SHR exhibit enhanced phosphorylation of ERK1/2 and AKT [4] that is implicated in enhanced proliferation [34]. To investigate if the overexpression of cell cycle proteins in VSMC from SHR is attributed to the enhanced activity of these kinases, the effects of PD 98059, a MAP kinase inhibitor and wortmannin, a PI3kinase inhibitor on the expression of cell cycle proteins were examined in VSMC from SHR. As shown in Figs. 7 and 8, PD 98059 (Fig. 7) as well as wortmannin (Fig. 8) significantly inhibited the enhanced expression of cyclin D1 (A), cdk2 (C) and pRb (D) in VSMC from SHR. These data suggest that the increased expression of cell cycle proteins, cyclin D1, cdk2 and pRB in VSMC from SHR is attributed to the enhanced activity of MAP kinase and PI3kinase.

### C-ANP_4–23_ inhibits the enhanced phosphorylation of ERK1/2 and AKT in VSMC from SHR

**Figure 9:**
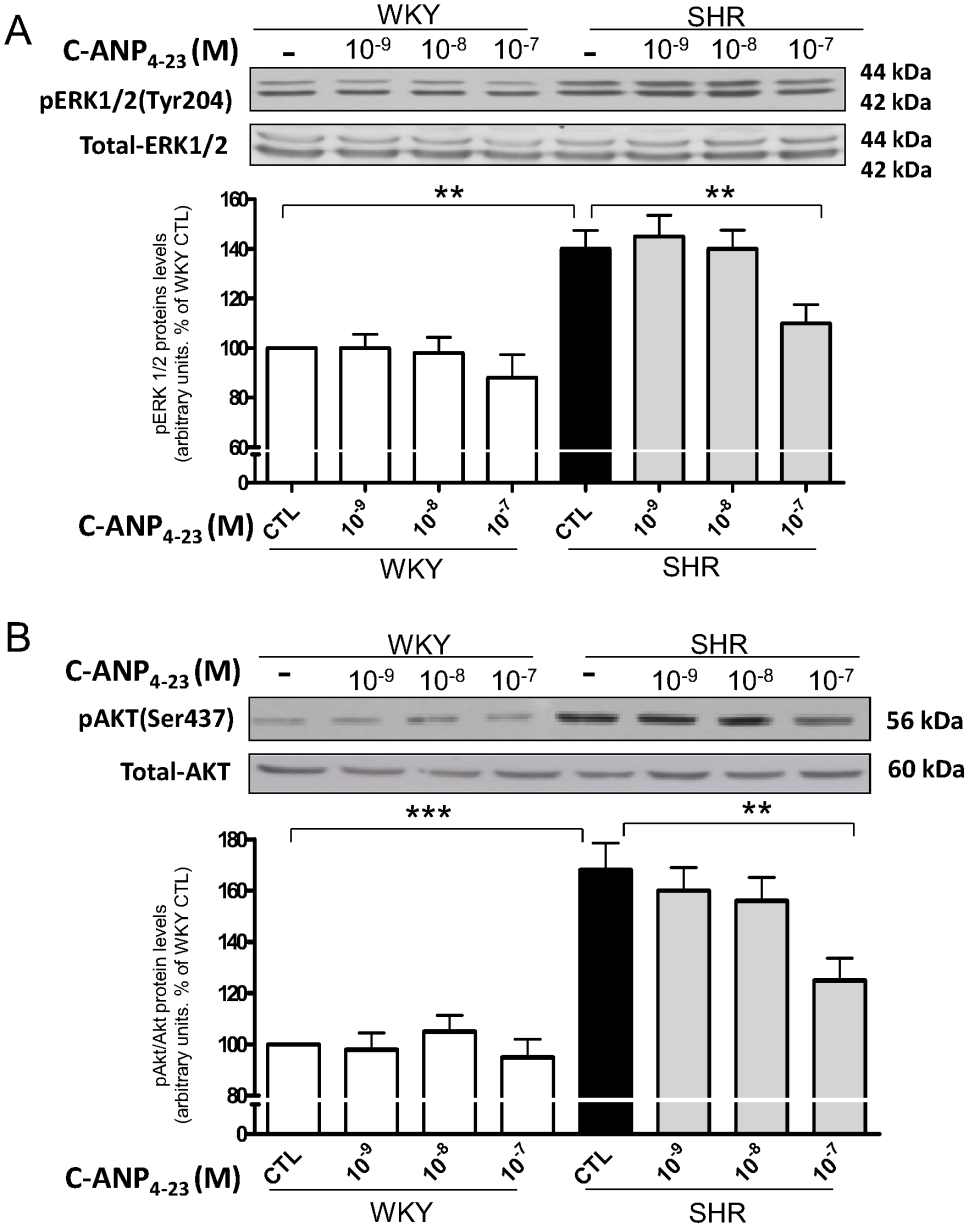
Effect of C-ANP_4–23_ on phosphorylation of ERK (A) and AKT(B) expression in vascular smooth muscle cells (VSMC) from SHR and WKY rats. VSMC from SHR and WKY rats were incubated in the absence (control) or presence of different concentrations of C-ANP_4–23_ for 16 h. The cell lysates were prepared and used for Western blotting using specific antibodies against pERK1/2 (A) and pAKT (B) as described in “Methods”. Results are expressed as % of WKY CTL taken as 100%. Values are means ± SE of 5 separate experiments. *P<0.05, **P<0.01.

To further investigate if C-ANP_4–23_-induced decreased expression of cell cycle proteins and resultant decreased proliferation is due to its ability to attenuate the enhanced activity of MAP kinase/PI3kinase, we examined the effect of C-ANP_4–23_ on the phosphorylation of ERK1/2 and AKT in VSMC from SHR. Results shown in Figure 9, indicate that the phosphorylation levels of ERK1/2 (A) as well as AKT (B) were increased by about 40% and 70% respectively in VSMC from SHR as compared to WKY and C-ANP_4–23_ at 10-^7^ M abolished the enhanced phosphorylation of ERK1/2 whereas the augmented phosphorylation of AKT was attenuated by about 60%. On the other hand, C-ANP_4–23_ at lower concentrations was ineffective in attenuating the enhanced phosphorylation of ERK1/2 or AKT.

## Discussion

VSMC from SHR in the presence of Ang II and FBS have been shown to exhibit exaggerated growth which was shown to be associated with progression from G1 to S phase [7], [8]. In addition, the expression of cell cycle proteins from G1-phase was shown to be up-regulated in VSMC from SHR [7], [9]. We recently reported that vasoactive peptide-induced increased proliferation of A10 VSMC was attenuated by small peptide fragments of cytoplasmic domain of NPR-C having Gi activator sequences [28]. In the present study, we show for the first time that the NPR-C agonist, C-ANP_4–23_ attenuates the enhanced proliferation of VSMC from SHR due to its ability to decrease the enhanced expression of cell cycle proteins cyclin D1, cdk2, cdk4 and pRb from G1-phase.

We report that the hyperproliferation of VSMC from SHR is attributed to the enhanced expression of cyclin D1, cdk4, cyclin A, cyclin E and cdk2 because the inhibitors, NSC 625987 and NU2058 of cyclin D1/cdk4 and cdk2 respectively decreased the enhanced proliferation of VSMC of SHR. In addition, the fact that the expression of phosphorylated form (pRb) of Rb but not Rb *per se* was significantly increased in VSMC from SHR as compared to VSMC from WKY suggest that the phosphorylated Rb is responsible to initiate the transition from G1 phase to S phase and that the activities of cyclin D1/cdk4 and cyclin E/cdk2 complexes may be enhanced in VSMC from SHR as compared to VSMC from WKY. Our results are consistent with the studies of Lee et al. who have also shown that VSMC from SHR exhibit enhanced activities of cdk2 and cdk4 kinase [9].

ANP has been shown to restrain G_1_-S cell cycle progression by a regulation of the expression of the cell cycle proteins in astrocytes [35]. However, in the present study, we show for the first time that in VSMC from SHR, NPR-C agonist, C-ANP_4–23_ decreased the enhanced expression of cyclin D1, cyclin A, cyclin E, cdk2 and cdk4. Furthermore, the fact that the phosphorylation of pRb protein is attenuated by C-ANP_4–23_ further supports that C-ANP_4–23_-evoked decreased activities of cyclin D1/cdk4 and cyclin E/cdk2 complexes may be responsible for the attenuation of enhanced proliferation of VSMC from SHR. Thus, taken together, it is suggested that the activation of NPR-C by C-ANP_4–23_ attenuates the enhanced proliferation of VSMC from SHR by decreasing the expression of cell cycle proteins.

The mechanism/s responsible for enhanced expression of cell cycle proteins in VSMC from SHR involve Giα proteins and MAP kinase/PI3kinase signaling. The fact that pertussis toxin attenuated the enhanced expression of cdk2, cdk4 cyclin D1 and enhanced phosphorylation of Rb to control levels suggest that the enhanced levels of Giα proteins in VSMC from SHR contribute to the enhanced expression of cell cycle proteins in VSMC from SHR. In this regard, the implication of enhanced levels of Giα proteins in enhanced proliferation of VSMC from SHR has also been demonstrated [4]. Furthermore, the fact that C-ANP_4–23_ also attenuated the enhanced expression of Giα proteins in VSMC from SHR suggests that C-ANP_4–23_-induced decreased expression of cell cycle proteins from G_1_ phase and resultant attenuation of hyperproliferation of VSMC from SHR is attributed to its ability to decreases the enhanced expression of Giα proteins. However, the activation of NPR-C by C-ANP_4–23_ and resultant decreased levels of intracellular cAMP [22], [23] may not be the underlying mechanism contributing to the antiproliferative effect of C-ANP_4–23,_ because the decreased levels of cAMP have been reported to cause hyperproliferation of the cells [36], [37]. This notion is supported by the studies of other investigators who have also shown that C-ANP_4–23_ inhibits cell proliferation by cAMP-independent mechanism [38].

Several studies have shown the implication of MAP kinase and PI3-kinase in cell proliferation [39], [40]. In addition, the hyperproliferation of VSMC from SHR was also shown to be attributed to the augmented activity of MAP kinase and PI3kinase [28], [34], [41]. We have earlier shown that small peptide fragments of cytoplasmic domain of NPR-C attenuated the vasoactive peptide-induced enhanced proliferation via MAP kinase/PI3kinase signaling in A10 cells [28]. However, in the present study, we show that C-ANP_4–23_ attenuated the enhanced phosphorylation of ERK1/2 and AKT in VSMC from SHR and suggest that the antiproliferative effect of C-ANP_4–23_ in VSMC from SHR is also mediated through the inhibition of MAP kinase/PI3-kinase/AKT signaling pathways. Furthermore, the fact that the enhanced expression of cyclin D1, cdk2 and pRb phosphorylation in VSMC from SHR was also attenuated by MEK inhibitor PD98059 and wortmannin, a PI3-kinase inhibitor, suggests the implication of MAP kinase and PI3-kinase in enhanced levels of cell cycle proteins in VSMC from SHR. Thus, taken together it is suggested that C-ANP_4–23_-mediated attenuation of MAP kinase and PI3kinase activation in VSMC from SHR contributes to the decreased expression of cell cycle proteins and thereby decreased cell proliferation.

In conclusion, we demonstrate for the first time that the enhanced expression of Giα proteins and MAPkinase/PI3kinase activation contribute to the enhanced expression of cell cycle proteins in VSMC from SHR. The activation of NPR-C by C-ANP_4–23_ inhibits the enhanced expression of cell cycle proteins in VSMC from SHR through its ability to inhibit the enhanced activation of MAP kinase and PI3K/AKT and enhanced expression of Giα proteins and result in the attenuation of hyperproliferation. From these studies, it can be suggested that C-ANP_4–23_ could be used as a therapeutic agent in the treatment of vascular complications associated with hypertension, atherosclerosis and restenosis.
